# Survey-calibrated agent-based modeling of peer targeting for promoting physical activity among adolescents

**DOI:** 10.3389/fpubh.2026.1807576

**Published:** 2026-06-04

**Authors:** Weixuan Long, Binbing Zheng, Zhihan Wu, Hongsheng Qian, Yu Zou, Jingwang Tan, Yilei Wang

**Affiliations:** 1 Department of Sport Science, College of Education, Zhejiang University, Hangzhou, Zhejiang, China; 2 College of Physical Education, Shanghai University of Sport, Shanghai, China; 3 College of Physical Education, Wuhan Sports University, Wuhan, Hubei, China; 4 College of Physical Education, Shanghai University, Shanghai, China; 5 Department of Sports, Hangzhou Medical College, Hangzhou, China

**Keywords:** adolescent, agent-based model, centrality-based peer targeting, physical activity, social network intervention

## Abstract

**Objectives:**

This study aims to compare the effectiveness of various centrality-based peer-targeting strategies in promoting physical activity (PA) among adolescents and identify practical principles for school-based social network interventions.

**Study design:**

A modeling study combining cross-sectional survey data with an agent-based model (ABM) analysis.

**Methods:**

Questionnaire data from 1,692 middle-school students were used to parameterize PA distributions, friendship ties, and co-activity probabilities. Maximum-entropy class networks were generated, and a peer-influence diffusion process inspired by the Susceptible–Infected–Recovered (SIR) framework was implemented. Four targeting strategies including random, degree-, betweenness-, and closeness-centrality were evaluated based on diffusion speed and changes in the proportions of low-, moderate-, and high-PA students.

**Results:**

Centrality-based strategies generally accelerated PA diffusion relative to random targeting, although differences among centrality rules were small. Closeness-centrality showed a more concentrated diffusion pattern, but no single rule consistently dominated across all outcomes. Specific advantages varied by outcome: degree-centrality tended to strengthen already active clusters, while betweenness-centrality showed limited advantage in improving PA composition. Across all strategies, over 60% of adolescents who remained low active had below-average friendship degrees, indicating persistent structural disadvantages.

**Conclusion:**

Centrality-based peer-targeting strategies offer advantages over random allocation in school-based PA interventions. However, their effects on PA composition were limited, and socially peripheral adolescents remained difficult to engage through peer influence alone. No single centrality rule should be interpreted as universally optimal. These findings suggest that network-informed peer targeting should be combined with low-threshold, teacher- or coach-supported opportunities to improve inclusiveness and overall effectiveness.

## 1 Introduction

Physical inactivity (PA) among children and adolescents remains a major global public-health concern. Recent WHO estimates indicate that approximately 81% of adolescents worldwide fail to achieve the recommended 60 min of daily moderate-to-vigorous physical activity (MVPA) (1). In China, nationally representative surveillance shows similarly low activity levels, with approximately 84% of students aged 6–17 years classified as insufficiently active, and average daily MVPA of school-aged youth remaining well below the recommended threshold (2, 3). Despite multisector efforts, a large number of school-based PA trials and policy reforms in high-income countries have rarely raised accelerometer-measured MVPA; even ambitious programs in the United Kingdom (4) and the United States (5) have produced typically modest and inconsistent gains. From a social-behavioral perspective, PA adoption and maintenance among adolescents display features of social reinforcement. Behavioral frameworks such as social cognitive theory and the theory of planned behavior emphasize peer norms, perceived support, and self-efficacy in shaping PA intentions and behaviors (6–9). Empirical social-network studies consistently show that adolescents are more active when their friends are active and supportive of PA and that friendship ties form a natural substrate through which PA-related norms and practices diffuse in school settings (8, 9). However, in real-world schools, such network-based PA promotion operates under practical implementation constraints, including limited teacher time, fixed class schedules, class-bounded peer interactions, incomplete knowledge of students’ friendship networks, and limited feasibility of individualized or resource-intensive programs (10–12).

Over the past two decades, social network interventions (SNIs) have progressed from early proof-of-concept trials to a general framework for leveraging peer influence in health behavior change: school-based randomized trials and controlled network experiments showed that peer-led nomination models can shift adolescent behaviors and that adoption is strengthened by repeated, multi-source exposure within clustered friendship structures (13, 14). Prior work has integrated these strands into a network-interventions framework that identifies actionable levers, including identifying influential peers, segmenting communities, and adjusting network structures (15). Subsequent field studies comparing practical seeding rules further demonstrated that whom one targets can measurably alter population-level diffusion (16, 17). Recent work on social network interventions for children’s and adolescents’ PA also highlights the importance of implementation process, replicability, applicability, and generalizability when translating network-based strategies into school settings (18). Therefore, under routine school constraints, the practical question is not simply whether peer influence can support PA promotion, but which centrality-guided targeting strategies can generate relatively efficient diffusion within feasible school routines (19, 20). However, most existing studies either focus on outcomes other than school-based PA or assume fully observed networks and idealized diffusion settings. The specific evidence gap addressed in this study is how different centrality-guided targeting strategies perform in school-level social networks structured by classroom organization, partial relational information, and routine implementation constraints (21–25).

To address this gap, we develop a survey-calibrated agent-based model (ABM) on maximum-entropy random class networks to compare alternative centrality-based peer-targeting strategies under typical school constraints (26, 27). Traditional social network analysis can identify structurally central students but fails to capture how targeting them alters behavioral diffusion over time. Regression approaches, although useful for estimating peer associations, do not extend to simulating how the choice of different intervention seeds would alter diffusion trajectories. In contrast, ABM enables a direct comparison of alternative “what-if” intervention scenarios, by explicitly representing individual students, friendship ties, and targeting rules. Recent ABM research on school-based PA network interventions further shows that network data sources and centrality-based selection rules can influence simulated intervention effects (28). In the model, we set initial behavioral states and peer-influence parameters using empirical data from Shanghai middle schools based on Physical Activity Questionnaire for Adolescents (PAQ-A) scores and friendship nominations and parameterize class networks by size and average degree. We then implement a Susceptible-Infected-Recovered (SIR) diffusion process with a SIR-inspired state-transition process on these networks, comparing degree-, betweenness-, and closeness-based seeding rules to a non-targeted baseline. We evaluate two implementation-relevant endpoints: diffusion speed (steps to reach a class-level PA target) and changes in population composition, operationalized as shifts in the shares of low-, moderate-, and high-PA students.

Therefore, this study aims to compare how degree-, closeness-, and betweenness-based peer-targeting strategies perform under realistic school constraints. We expected centrality-guided targeting to improve diffusion efficiency relative to random targeting, but treated the relative advantages of different centrality rules as exploratory because their effects may vary across diffusion speed and PA composition outcomes.

## 2 Methods

This section explains how we analyze school survey data to anchor parameters and build an ABM to compare targeting strategies for adolescent PA. We first summarize sampling, measures, and statistics and make explicit how survey variables map to model inputs. Then, we specify the diffusion mechanism, network structure, and policy parameters used for simulation.

### 2.1 Empirical data and analysis

We conducted a stratified cluster sampling survey by randomly selecting six public middle schools (two suburban and four urban) in Shanghai. Within each school, 2–3 intact classes per grade (Grades 7–9) were randomly chosen, yielding 1719 completed questionnaires administered online and on paper. After excluding responses with missing key variables, 1,692 students (877 boys and 815 girls; 1,018 paper and 674 electronic) remained for analysis. PA was assessed with the Chinese version of the PAQ-A developed by Li et al., which has shown good internal consistency (Cronbach’s *α* > 0.8) and test–retest reliability > 0.8 in Chinese adolescents (29). The PAQ-A yields a 1–5 composite score reflecting typical MVPA during the previous 7 days. Following established cut-points, PA levels were categorized as low (≤2), moderate (>2 and <3), and high (≥3). These categories were used to summarize sex- and grade-specific baseline PA distributions and to initialize agents’ activity states in the simulation model. To capture students’ social context, the survey also included a peer nomination module. Students nominated close peers within the same grade, with no gender restrictions and up to 20 nominations permitted. For analyses of co-activity with peers, we focused on the five closest nominees to approximate the most influential friendship ties. The reported co-activity probability was used as an empirical reference for calibrating peer-influence parameters, rather than as a direct per-step transition probability. These peer nomination data were used to derive network characteristics, including the average number of friends per student. In the simulation, stochastic school-level networks were generated by representing students as nodes and assigning friendship ties to match the observed network size and average degree. These networks, together with co-activity probabilities, were used to calibrate peer influence and agents’ transition thresholds in the ABM. We conducted descriptive statistics and independent-samples t-tests, χ^2^ tests, and one-way ANOVA (with LSD *post hoc* comparisons) to profile sex- and grade-specific PA distributions, co-activity with friends, and network-related indicators. Descriptive statistics are summarized in Supplementary Table 1.

### 2.2 Agent-based modeling and simulation

This section describes the agent-based simulation used in this study. The model is implemented in NetLogo to evaluate school-feasible node-selection strategies under unified settings.

#### 2.2.1 SIR-inspired state-transition process for peer-influenced PA diffusion

The classic SIR model describes transitions among susceptible (S), infectious (I), and recovered (R) states in infectious-disease diffusion. In this study, we used this structure only as a conceptual analogy for peer-influenced PA diffusion, because behaviors and health-related states can also spread through social contacts, although not in the same way as pathogens. Previous studies have shown that exercise and PA can be socially influenced through peer exposure, social norms, encouragement, and co-activity opportunities (7, 30, 31).

In our SIR-inspired model, S does not indicate epidemiological susceptibility. Instead, it refers to students with low or insufficient PA who may be influenced by active peers. I refers to students who are currently active and can temporarily provide PA-related encouragement, modeling, or co-activity opportunities to connected peers. R is not treated as a permanent immune state; because adolescent PA may decline when reinforcement is absent, active students can return to a lower-activity state. Thus, the model operates as an S → I → S-style process rather than a conventional disease-based S → I → R process.

At each simulation step, active students exert influence on their connected peers. A lower-activity student may move to a higher PA state when peer exposure provides sufficient effective reinforcement during a simulation update, with this reinforcement level calibrated from baseline PA level and co-activity tendency. If social reinforcement is insufficient, an active student may return to a lower-activity state. This return process is a simplified behavioral assumption and should not be interpreted as a complete model of habit formation or long-term behavioral maintenance. This simplified state-transition process was used to compare how different peer-targeting strategies affect PA diffusion under the same network and parameter settings. The state-transition scheme is illustrated in Supplementary Figure 1.

#### 2.2.2 Structure and parameters

Model parameters were calibrated using questionnaire data and intervention scenario assumptions. The simulated network was designed to represent a school-level intervention setting. Baseline PA scores and PA-level distributions were derived from the questionnaire data. The reported co-activity probability was used as an empirical reference to derive the calibrated per-step peer-influence process, rather than being treated as the direct transition probability. Network generation used aggregate peer-nomination statistics, especially network size and average degree, to construct stochastic school-level networks. One simulation step represented one iteration of peer interaction and behavioral updating, rather than a fixed unit of real-world time. The simulation was terminated when the population-level mean PA score reached 2.75, corresponding to the PAQ-A cut-off for achieving ≥60 min of MVPA per day (32). Detailed empirical sources, calibration rules, and parameter values are provided in Supplementary Tables 2, 3. To assess robustness, we further varied the PA target threshold, initial seed proportion, and peer influence probability within plausible ranges and compared the number of simulation steps required to reach the population-level PA target across targeting strategies.

### 2.3 Centrality-based intervention strategies

**Figure 1:**
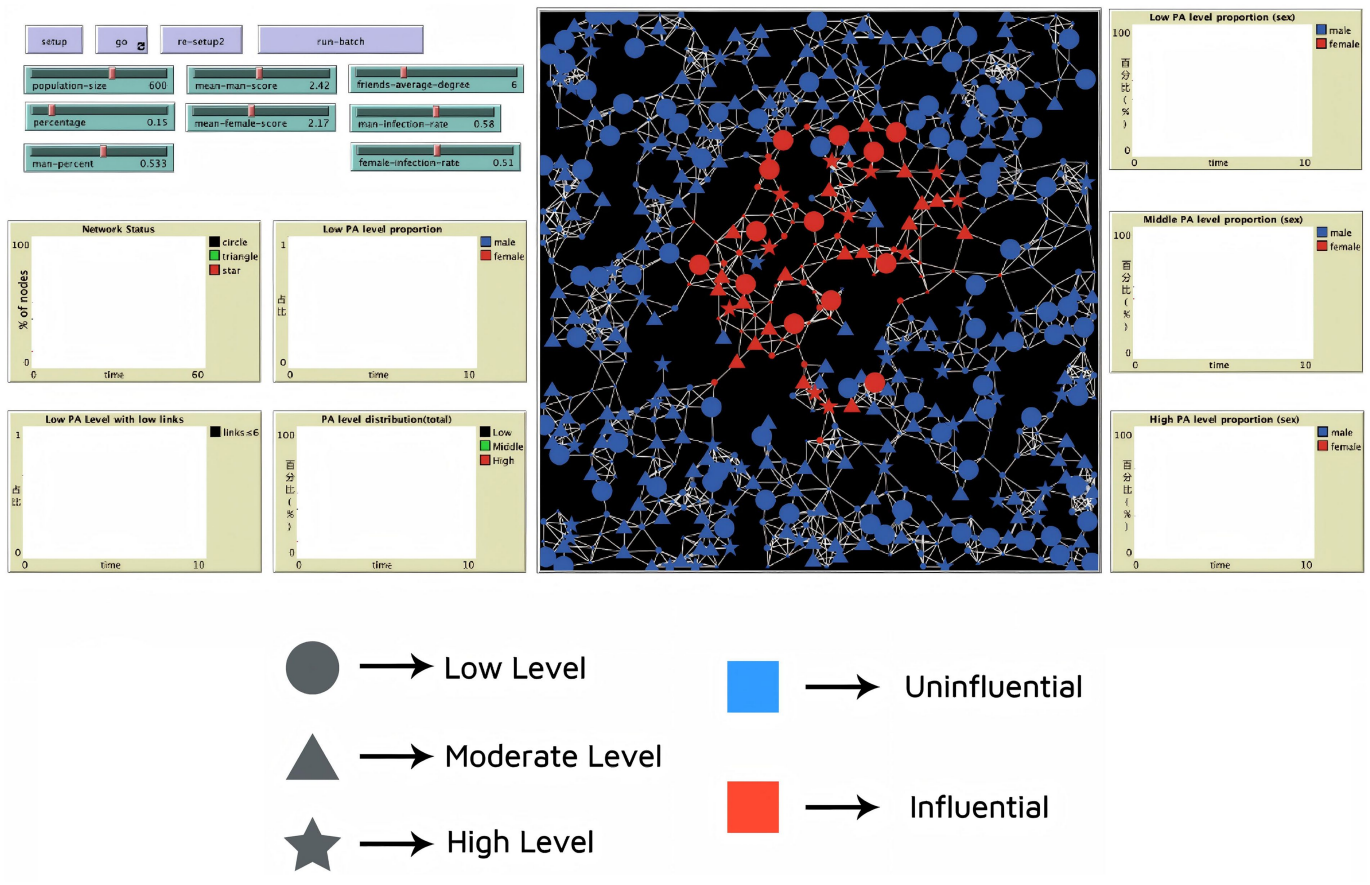
Agent-based model (ABM) simulation interface and agent diagram.

We implemented three centrality-guided seeding rules that capture different ways a student can be influential in a school network. Degree-centrality selects highly connected hubs with many direct ties to classmates. Closeness-centrality selects students who are, on average, only a few steps from everyone else, emphasizing fast reach. Betweenness-centrality selects bridge students that lie on the shortest paths between subgroups, emphasizing cross-group integration. Together, these strategies represent coverage (degree), speed (closeness), and bridging across cliques (betweenness) as complementary levers for seeding behavior change in schools (Figure 1).

## 3 Results

### 3.1 Questionnaire data analysis

#### 3.1.1 Gender and grade differences in PA

Boys were more active than girls. They had higher mean PAQ-A scores (2.42 vs. 2.17; *t* = 7.40, *p* < 0.001; Hedges’ *g* = 0.35), and the distribution across PA levels differed significantly by gender [*χ*^2^(2) = 47.83, *p* < 0.001; Cramér’s *V* = 0.17], with boys more often classified into the moderate and high PA groups and girls over-represented in the low PA group. Across grades, PAQ-A scores declined from Grade 7 to Grade 9 [*F*(2, 1,689) = 9.08, *p* < 0.05], accompanied by an increase in the proportion of students in the low PA category. Grade 9 students showed the lowest PA and the highest prevalence of insufficient activity (Supplementary Tables 4, 5).

#### 3.1.2 Gender and grade differences in social networks

Gender differences also emerged in social interactions. Relative to girls, boys nominated more friends (6.37 vs. 5.05; *t* = 6.09, *p* < 0.001; Hedges’ *g* = 0.29) and reported a higher probability of exercising with friends (0.59 vs. 0.51; *t* = 6.47, *p* < 0.001; Hedges’ *g* = 0.32). By contrast, grade differences in friend count [*F*(2, 1,689) = 1.49, *p* = 0.23] and co-activity probability [*F* (2, 1,689) = 0.38, *p* = 0.69] were small and non-significant (Supplementary Table 6).

#### 3.1.3 Correlations between PA and social interactions

To examine the link between PA and peer relations and to inform the ABM, we conducted Pearson and Spearman correlation analyses. As shown in Table 1, the number of nominated friends and the probability of exercising with friends were positively associated with PAQ-A scores and PA levels (*r* = 0.23–0.47, all *p* < 0.001). Although the association was weak (*r* = 0.225, *p* < 0.001), the two social indicators-friend count and co-activity probability-also showed a positively correlation. These results suggest that adolescents with more friends and a higher likelihood of exercising with them typically have higher PA, supporting the assumption that PA can diffuse along friendship ties and justifying the inclusion of PA level, friend count, and co-activity probability as key ABM parameters.

**Table 1:** Correlation analysis between social interaction and PA.

Variable	Pearson correlation coefficient/Spearman correlation coefficient			
	Number of friends	Probability of exercising with friends	PAQ-A score	PA level
Number of friends	1^**^			
Probability of exercising with friends	0.225^**^	1^**^		
PAQ-A score	0.255^**^	0.468^**^	1^**^	
PA level	0.234^**^	0.437^**^	0.906^**^	1^**^

**indicates *p* < 0.01. Pearson correlation analysis was used for the number of friends-probability of exercising with friends, number of friends-PAQ-A score, and probability of exercising with friends-PAQ-A score. Spearman correlation analysis was used for PA level-number of friends, PA level-probability of exercising with friends, and PA level-PAQ-A score.

### 3.2 Model stability check

In Supplementary Table 7, simulations were conducted on 100 independently generated random networks under identical statistical constraints. Across these network realizations, random targeting generally required more steps and showed greater between-run variability than the three centrality-based strategies. On average, targeting structurally central students accelerated PA diffusion across different network structures. These findings suggest that diffusion outcomes vary with underlying network structure and provide a structural-level stability check for the model. However, this comparison is not intended to constitute a full parameter-based sensitivity analysis.

### 3.3 Sensitivity analysis

Sensitivity analyses were conducted to examine whether the main findings were robust to plausible variations in key model parameters. As shown in Supplementary Table 8, changes in the PA target threshold, initial seed proportion, and peer influence probability affected the absolute number of simulation steps required to reach the PA target, but did not alter the overall pattern of results. Across the tested scenarios, centrality-based targeting strategies generally required fewer steps than random targeting. However, the differences among degree-, closeness-, and betweenness-centrality strategies were relatively small, and no single centrality rule consistently dominated across scenarios. These results indicate that structurally informed peer targeting generally accelerated PA diffusion relative to random selection under moderate parameter variations.

### 3.4 Simulation results of targeting strategies in the model

**Figure 2:**
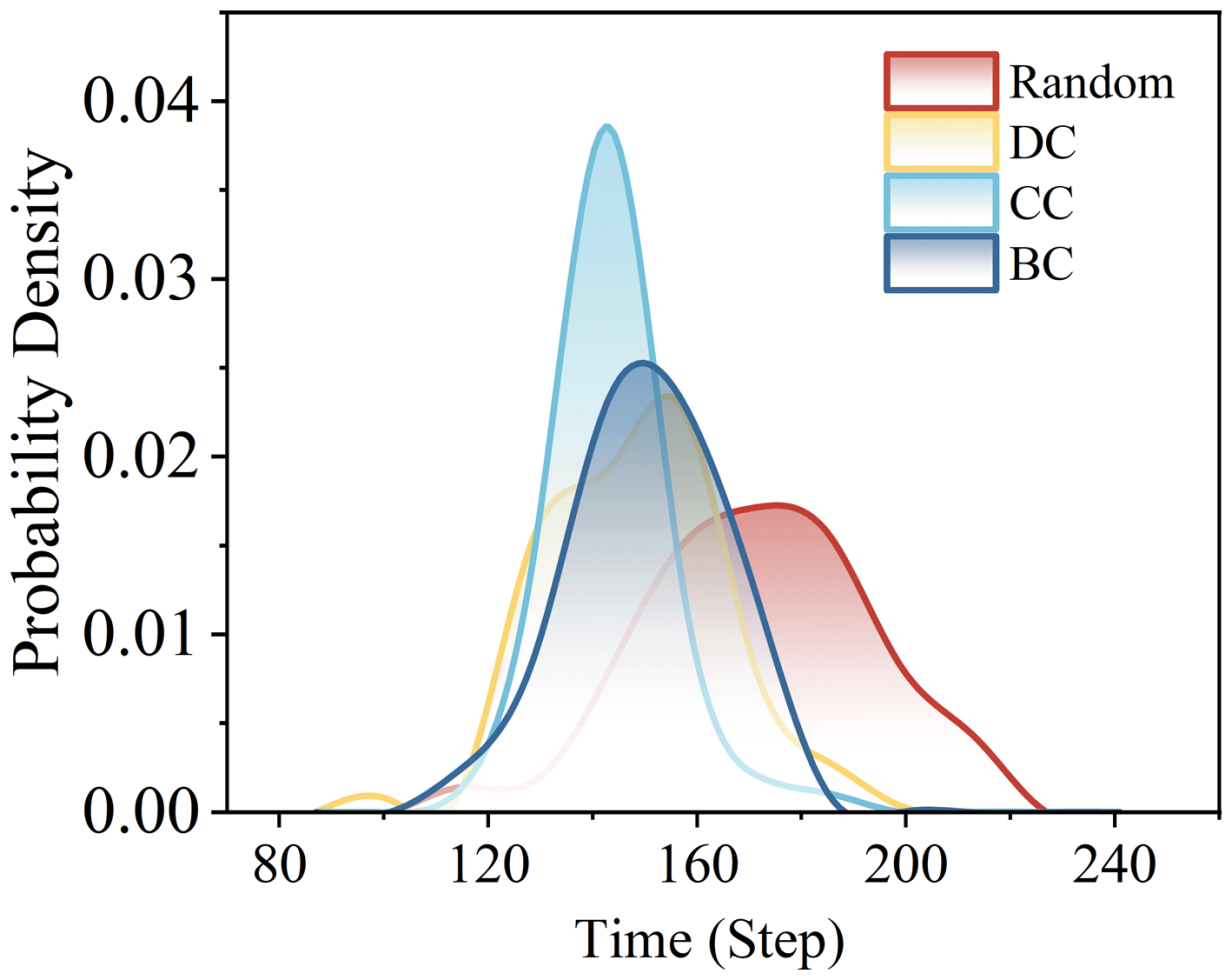
Probability density functions of step counts under four targeting strategies. Targeting strategies are defined as follows: Random: random targeting; DC, degree-centrality; CC, closeness-centrality; BC, betweenness-centrality.

Figure 2 presents the probability density distributions of the number of simulation steps required to reach the PA target under the four targeting strategies. The random strategy produced the slowest and most variable diffusion, with a peak around 171 steps. The three centrality-based strategies shifted the distributions toward faster diffusion, with partly overlapping distributions. Degree-centrality showed a peak around 157 steps, while closeness-centrality showed an earlier peak around 143 steps with a relatively concentrated distribution. The peak and dispersion of the betweenness-centrality strategy fell broadly within the range of the other two centrality-based strategies. Overall, these patterns suggest that centrality-based targeting mainly improved diffusion efficiency compared with random targeting, but differences among centrality rules should be interpreted cautiously.

**Figure 3:**
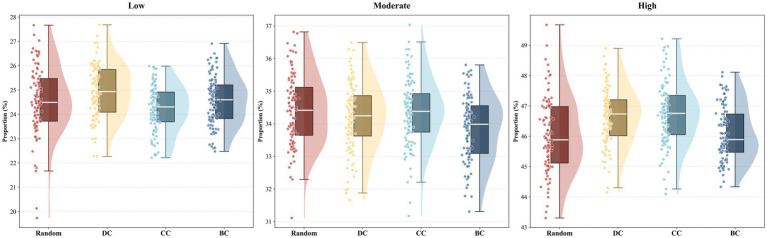
Comparison of proportions of high-, moderate-, and low-PA levels across four targeting strategies.

**Figure 4:**
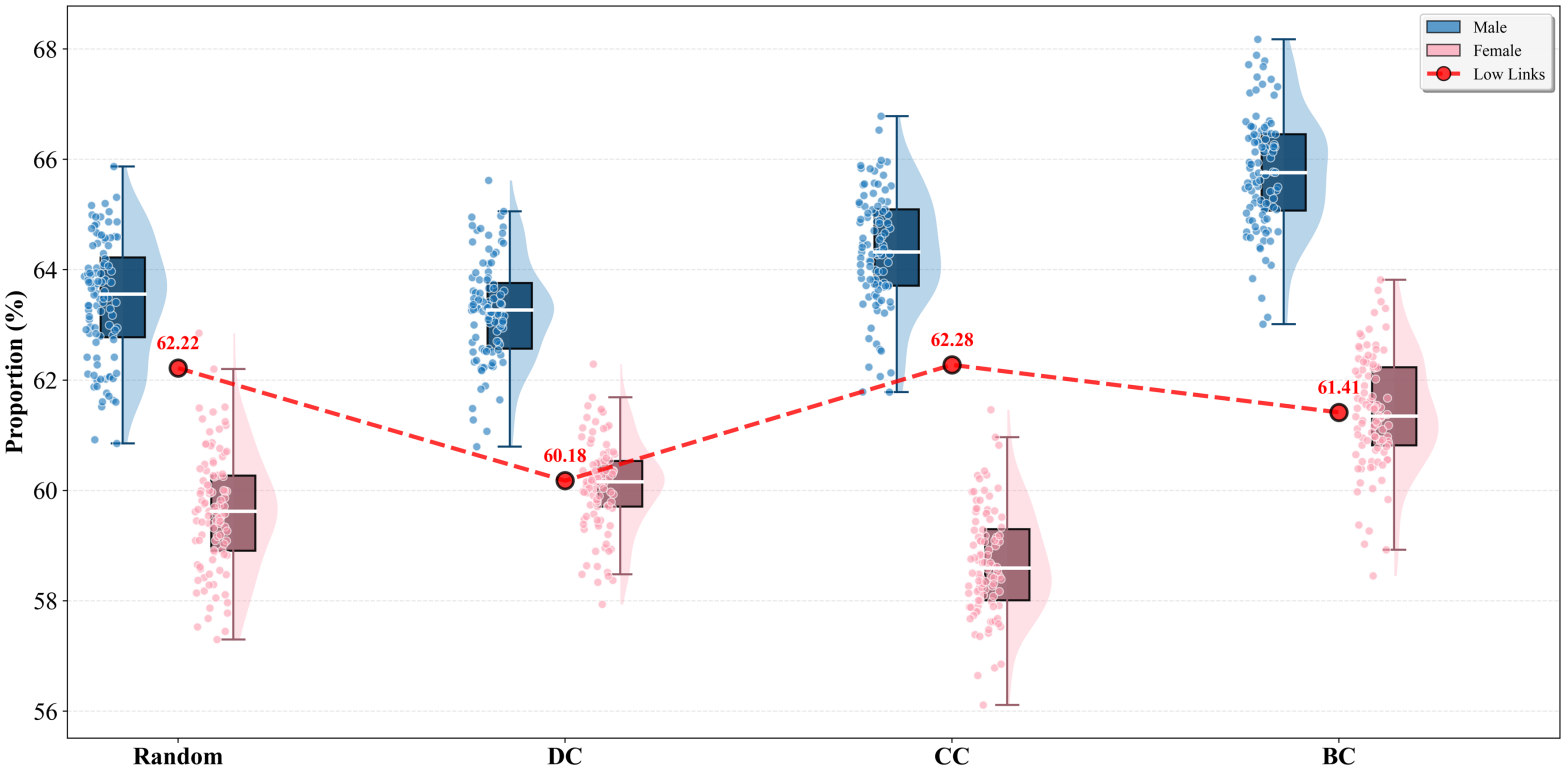
Unequal reduction of low-PA students by sex across targeting strategies.

Building on these diffusion patterns, Figures 3, 4 summarize how the four targeting strategies reshaped the final composition of low-, moderate-, and high-activity students, plus the structural characteristics of the low-activity group. All four strategies converged to a final low-activity proportion of roughly 24–25%. Under the random strategy, the mean was 24.55% (median = 24.49%, range = 19.73–27.67%) with a relatively dispersed distribution. Closeness-centrality strategy yielded a slightly lower mean of 24.24% (median = 24.31%, range = 22.21–25.98%) and a narrower range, indicating a modest improvement and more concentrated outcomes. By contrast, degree-centrality targeting produced the highest mean (24.95%), while betweenness-centrality targeting’s mean (24.56%) was nearly indistinguishable from the random strategy, offering little advantage in reducing the low-activity subgroup. In the moderate-activity group, the four strategies yielded very similar final proportions (range: 31.11–37.03%). This pattern suggests that targeting strategy had minimal influence on this subgroup, with main compositional shifts occurring at the two ends of the activity spectrum. The high-activity group showed the reverse pattern of the low-activity group. Random strategy had a mean of 46.03% (median = 45.88%). Closeness-centrality targeting increased the mean slightly to 46.74% (median = 46.76%, range = 44.10–49.21%), while degree-centrality targeting yielded a very similar mean (46.66%, range = 44.15–48.90%). Betweenness-centrality targeting again showed no clear advantage (mean = 46.09%, range = 44.33–48.11%), only marginally higher than random. Combined with the diffusion-speed results, these compositional outcomes suggest that centrality-based targeting mainly improved diffusion efficiency, whereas changes in PA composition were modest. The apparent advantage of closeness-centrality was more evident in diffusion concentration than in large shifts in PA distribution, and betweenness-centrality should be interpreted cautiously rather than dismissed as ineffective.

To further characterize who remained low active under each targeting strategy, we examined sex-specific patterns and students’ network positions. The sex-stratified analysis showed that boys were more likely than girls to remain in the low-activity group after diffusion. Across all four strategies, the final proportion of boys who remained low active mostly fell between 63.25 and 65.76%, whereas the corresponding proportion for girls mostly ranged from 58.69 to 61.41%. Meanwhile, the red dashed line in Figure 4 shows that, under every targeting strategy, more than 60% of the students who remained low active had below-average friendship degrees. This indicates that structurally peripheral adolescents, those with fewer friendship ties, constitute the majority of the persistently low-active group regardless of which targeting strategy is applied.

## 4 Discussion

Using survey-calibrated agent-based simulations, this study examined how three centrality-based targeting rules influence the speed and distributional outcomes of PA diffusion in typical middle-school friendship networks. While all targeted strategies outperformed random selection, they diverged in balancing rapid diffusion, stability, and shifts in low-, moderate-, and high-PA groups. These differences highlight the need to further explore (1) why relative advantages varied across outcomes, (2) why no single centrality rule consistently dominated, and (3) why structurally peripheral low-PA adolescents remain hard to reach. Accordingly, we integrate empirical evidence and network theory to interpret these patterns, discussing sex differences, practical implications for intervention design, and methodological strengths and limitations.

### 4.1 Interpretation of questionnaire findings and their alignment with existing research

The questionnaire findings indicated clear social clustering of PA. Highly active students tended to exercise with friends, whereas low-active students were often located at the network periphery with fewer opportunities for joint activity and social reinforcement. This pattern is consistent with evidence of peer homophily, social dependence, and imitation in adolescent PA (33–35). The observed gender and grade patterns also align with previous research. Girls report lower MVPA and weaker PA self-efficacy than boys (36, 37), and increasing academic pressure in higher grades is associated with lower participation (38). However, students with sparse friendship ties and limited involvement in group activities appear structurally disadvantaged in receiving social reinforcement, echoing earlier network studies of youth PA (39, 40). Taken together, these results show that PA behavior is deeply embedded in school peer networks and provide a structural explanation for the unequal influence of different targeting strategies in our simulations.

### 4.2 Mechanistic interpretation of simulation results

Across strategies, our simulations indicate that centrality-based targeting generally accelerated PA diffusion relative to random selection, although the differences among centrality strategies and the shifts in PA distribution were modest. These patterns can be interpreted within the SIR-inspired peer-influence framework introduced earlier (14, 24), in which PA diffuses through adolescent friendship networks. The transition process in the model focuses on social-network exposure and peer reinforcement, while other determinants of PA, such as habit formation, motivation, disability or medical limitations, facility access, and family support, are not explicitly modeled. Therefore, the results mainly compare the relative performance of different network-based targeting rules under shared assumptions.

Students with high closeness-centrality occupy positions that minimize average path lengths to many peers and classes, which may help PA-related norms reach multiple clusters relatively quickly and with broad coverage. Prior network experiments and ABM studies have shown that reducing distances between communities and expanding multi-cluster reach can be more decisive than simply increasing local degree when spreading behaviors that rely on social influence (16, 27). This interpretation is consistent with earlier simulations in Dutch classrooms, where closeness-based targeting showed favorable performance for increasing average activity levels (27). In the present study, closeness-centrality remained theoretically meaningful because it produced a relatively concentrated diffusion pattern, but it should be interpreted as one plausible strategy rather than a universally superior rule.

Under the present model assumptions, degree-centrality targeting appeared to mainly strengthen already active or moderately active clusters, with only marginal additional reductions in low-PA students compared to random selection. This pattern is consistent with a local “broadcast” mechanism: highly connected students can rapidly diffuse PA information and norms within their immediate friendship circles, which are often composed of classmates who already have moderate or high PA or strong interest in sport (35, 39). As highlighted in reviews of network composition and centrality, such strategies tend to reinforce existing advantages in well-connected cores rather than transform the overall distribution (41). In practice, degree-based rules may be more suitable when the goal is to consolidate or stabilize an existing PA culture, although they may be less effective for reaching low-PA adolescents under similar network conditions.

Betweenness-centrality targeting did not show a stable advantage across outcomes, suggesting that bridge positions alone may not reliably drive PA change in this context. Many high-betweenness students may connect groups through relatively weak or low-frequency ties with limited joint activity, which may be insufficient when bridging ties do not correspond to PA-related reinforcement. Moreover, some bridge actors may not be perceived as credible PA models, which could limit their normative influence across clusters. This contrasts with the gender-specific ABM by Zhang et al. (42), who reported that betweenness-based targeting was particularly effective for promoting weight-related healthy behaviors among girls in a small-world school network. Differences in behavioral outcomes (general health and weight-related behaviors vs. PA levels), network construction (small-world vs. maximum-entropy class networks), and calibration (gender-specific diffusion parameters vs. class-bounded, academically structured networks) may all contribute to this discrepancy, together with contextual factors in Chinese school timetables that restrict cross-class opportunities for joint PA. Taken together, our results suggest that structurally informed targeting can modestly improve diffusion efficiency compared with random selection under the present model assumptions. However, the choice among centrality rules should be cautious because the differences were small and context-dependent. No single centrality rule should be interpreted as universally optimal for PA promotion.

### 4.3 Students left behind: structural inequities in diffusion

A substantial subset of adolescents who began with low PA levels showed little improvement under any targeting strategy. Many of these students had below-average friend counts and occupied peripheral positions in the network, which is consistent with evidence that adolescents with fewer or less active friends are less likely to engage in PA and are less responsive to peer-focused approaches (36, 43). From a complex-contagion perspective, sparse local networks provide too little repeated, multi-source reinforcement to support durable behavior change, especially for activities that require effort and coordination (6). In our simulations, girls were only slightly more responsive than boys. Chinese middle school students tend to have cohesive class networks and similar academically oriented peer groups for both sexes, which may dampen gender differences in diffusion (31). Collectively, these findings suggest that centrality-based targeting is necessary but not sufficient for an equity perspective. Such strategies can accelerate diffusion among moderately embedded students, but structurally under-connected adolescents remain difficult to engage, regardless of sex. This aligns with a review showing that peer-network interventions are most effective when combined with broader changes to the school environment and additional adult-led support (44). In practice, data-driven peer targeting should therefore be complemented by low-threshold, teacher- or coach-led opportunities, such as small-group activity clubs or inclusive, non-competitive programs that are explicitly designed to draw in students on the social and behavioral periphery.

### 4.4 Practical implementation implications

In real school settings, complete social-network mapping is rarely feasible, so a practical question is how to identify “key students” using information that teachers already have. Our simulations, alongside prior work on adolescent networks, suggest several observable traits can serve as simple heuristics: students who interact frequently across classes, are visible in grade-level activities, participate in multiple clubs or sports teams, and are widely recognized by teachers and peers as socially connected are more likely to occupy central positions in friendship networks. Schools can start from these candidates and then refine choices using local knowledge about students’ willingness, workload, and equity concerns, thereby designing network-informed PA interventions that remain feasible in everyday practice.

## 5 Strengths and limitations

This study has several methodological strengths. First, it tightly links large-sample survey data with an agent-based social network model. To enhance ecological validity and interpretability of simulated diffusion, we directly used empirical PA distributions, peer nominations, and co-exercise probabilities to initialize agent states, calibrate peer-influence parameters, and approximate network density. Second, by embedding an SIR-inspired peer-influence process within the ABM, we examined two implementation-relevant outcomes, diffusion speed and shifts in the proportions of low-, moderate-, and high-PA students, rather than focusing only on final coverage. Third, we incorporated sex-specific behavioral differences from the survey into agent configurations, allowing the model to better reflect gendered PA patterns and peer interactions in real schools. Together, these strengths provide a modeling framework adaptable to testing alternative peer-targeting strategies in other school environments.

Several limitations should also be noted. The survey was cross-sectional and conducted at a single time point, so we cannot assess temporal changes in friendships or PA; longitudinal social network data will be needed to validate the stability of key parameters. In addition, the study was conducted in Shanghai, a highly urbanized city with relatively developed school resources, dense academic schedules, and structured class-based organization. These features may differ from schools in smaller cities, rural areas, or regions with different extracurricular activity systems and peer-network patterns, which may limit the generalizability of the findings beyond this context. Simulated networks were maximum-entropy random graphs calibrated only by class size and degree, omitting clustering, community structure, and overlapping subgroups, which may distort diffusion pathways and lead to either overestimation or underestimation of peer influence effects in structured real-world networks. Behavioral parameters such as transition and recovery probabilities, although informed by empirical distributions, still involve simplifying assumptions and subjective choices. Although sensitivity analyses supported the robustness of the main findings under moderate parameter variation, only a limited set of parameters was examined. Finally, the ABM includes a limited set of agent attributes and simplifies the behavioral transition process. Although baseline PA, sex-specific PA patterns, friendship ties, and co-activity probability were used to introduce empirical heterogeneity, the model focuses primarily on social-network exposure and peer reinforcement, and does not explicitly capture long-term habit formation, disability or medical limitations, facility access, family support, classroom climate, school policies, or time-varying psychological factors. Accordingly, the “realistic school constraints” considered here represent a feasibility-oriented implementation setting rather than a complete account of all determinants of adolescent PA. Future studies should extend the model by incorporating richer individual constraints, longitudinal PA data, and history-dependent transition rules.

## 6 Conclusion

This study combined school survey data with an agent-based social network model to examine how centrality-based targeting strategies shape PA diffusion among middle-school students. Across simulations, centrality-guided targeting generally accelerated diffusion relative to random selection, but the differences among centrality strategies and the changes in PA composition were modest. No single centrality rule dominated across all outcomes, although different rules showed context-dependent advantages. These findings suggest that centrality-based peer targeting should be understood as a modest efficiency-enhancing strategy rather than a stand-alone solution for substantially increasing adolescent PA. Peer-led diffusion should therefore be complemented by teacher-supported, inclusive, and low-threshold opportunities for students who remain socially or behaviorally peripheral. Future studies should use longitudinal social network data, observed school networks, and richer behavioral assumptions to test the stability and generalizability of these findings.

## Data Availability

The raw data supporting the conclusions of this article will be made available by the authors, without undue reservation.
