# Supplementary material for: Survey-calibrated agent-based modeling of peer targeting for promoting physical activity among adolescents
Source: Front Public Health. 2026 Jun 4;14:1807576. doi: 10.3389/fpubh.2026.1807576 (PMC13292596; doi:10.3389/fpubh.2026.1807576)
Supplement: Supplementary file 1 [file Data_Sheet_1.docx]

Supplementary Material

# Supplementary Figures and Tables

## Supplementary Figures


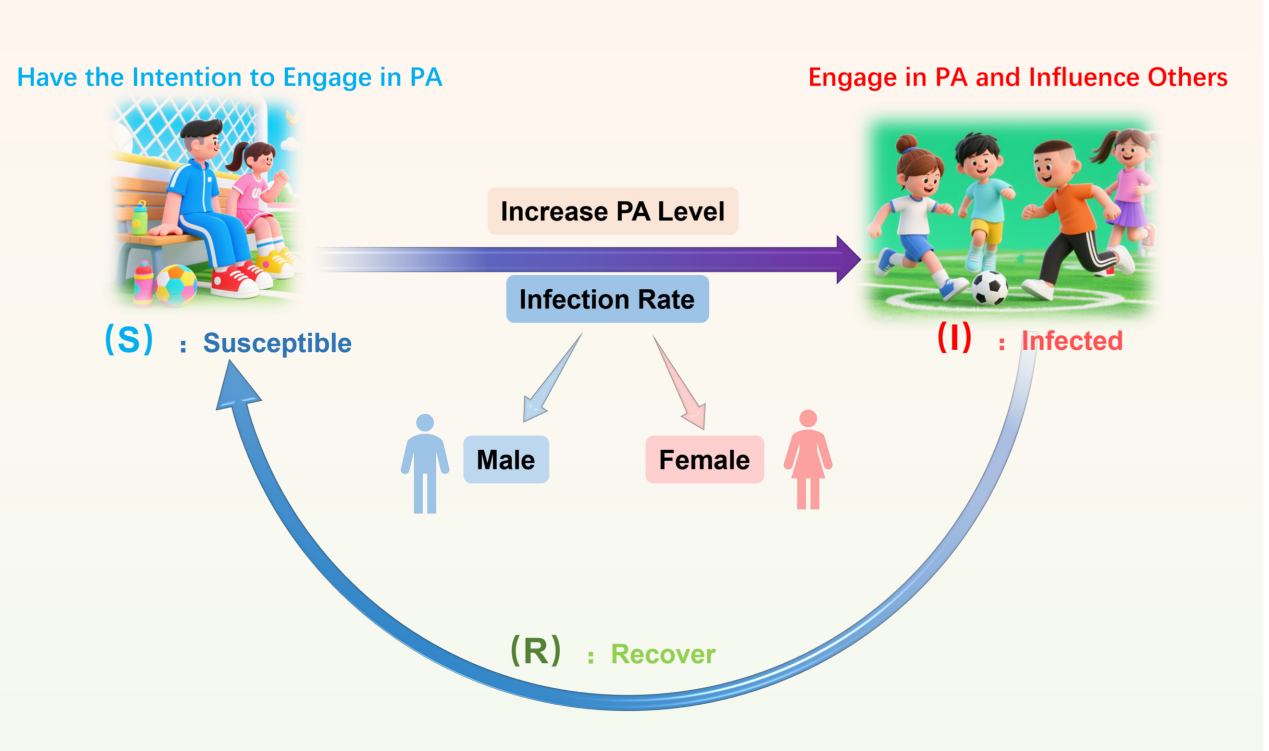


**Supplementary Figure 1.** SIR-inspired state-transition process for peer-influenced PA diffusion.

## Supplementary Tables

**Supplementary Table 1.** Data collection and descriptive analysis of adolescents survey.

|  |  | Number  of student | Proportion | Mean ± SD |
| --- | --- | --- | --- | --- |
|  | Male | 877 | 51.83% | / |
| Gender |  |  |  | / |
|  | Female | 815 | 48.17% |  |
|  | 7 | 561 | 33.15% | / |
| Grade | 8 | 874 | 51.65% | / |
|  | 9 | 257 | 15.20% | / |
|  | Low | 632 | 37.40% | 2.30 ± 0.70 |
| PA-level | Moderate | 848 | 50.10% |  |
|  | High | 212 | 12.50% |  |
|  |  |  |  |  |
| Number of friends |  |  |  | 5.73 ± 4.57 |
|  |  |  |  |  |
| Probability of exercising with friends |  |  |  | 0.549 ± 0.2 |

**Supplementary Table 2.** Agent-based model parameters, empirical sources, calibration rules, and model roles

| Model parameter | Meaning in the ABM | Empirical source / assumption | Calibration or assignment rule | Baseline value | Sensitivity range | Model role |
| --- | --- | --- | --- | --- | --- | --- |
| Population size | Number of agents in the simulated network | Intervention scenario assumption | Set to represent a school-level simulation scenario | 600 | NA | Network scale |
| Sex ratio | Male/female composition | Student survey | Initialized according to empirical sex distribution | Male: 51.83%; Female: 48.17% | NA | Agent attribute |
| Baseline PA score | Initial PA tendency | PAQ-A survey | Assigned according to sex-specific PAQ-A mean and PA-level distribution | Male: 2.42; Female: 2.17 | NA | Initial agent state |
| PA-level distribution | Low-, moderate-, and high-PA composition | PAQ-A classification | Low ≤2;  moderate >2 and <3;  high ≥3 | 37.40%;  50.10%;  12.50% | NA | Initial state distribution |
| Friendship degree | Average number of friendship ties | Peer nomination survey | Rounded from empirical mean of 5.73 | 6 | NA | Network density |
| Raw co-activity probability | Reported tendency to exercise with friends | Survey item | Used as empirical reference for peer-influence calibration | Overall: 0.549; Male: 0.585; Female: 0.510 | NA | Empirical calibration reference |
| Calibrated per-step peer-influence process | Effective peer influence during one simulation update | Derived from raw co-activity probability | Raw co-activity probability was used to derive, but not directly equal, the per-step transition process | Calibrated setting | ±10% | Transition process |
| Transition condition | Condition for moving to a higher PA state | Model assumption informed by PA level and co-activity tendency | Peer exposure must provide sufficient effective reinforcement during one update | Category-specific | NA | State transition |
| Recovery / decline process | Return to lower PA state when reinforcement is insufficient | Behavioral simplification | Active state may decline when reinforcement is not sustained | Model setting | NA | Return process |
| Initial seed proportion | Proportion of initially targeted active agents | Intervention scenario assumption | Top-ranked students selected according to each targeting strategy | 0.15 | 0.12–0.18 | Intervention intensity |
| Targeting strategy | Rule for selecting initial seeds | Simulation design | Random, degree-centrality, closeness-centrality, and betweenness-centrality | Four strategies | NA | Intervention scenario |
| PA target threshold | Population-level stopping criterion | PAQ-A cut-off literature | Simulation stops when population-level mean PAQ-A reaches target | 2.75 | 2.65–2.85 | Outcome criterion |
| Simulation step | One iteration of behavioral updating | Model assumption | One round of peer exposure and agent-state update | NA | NA | Time unit |

**Supplementary Table 3.** Calibration consistency check between empirical targets and model initialization values

| Calibration target | Empirical survey value | Model initialization value | Difference | Calibration interpretation |
| --- | --- | --- | --- | --- |
| Male proportion | 51.83% | 51.83% | 0.00 pp | Directly initialized |
| Female proportion | 48.17% | 48.17% | 0.00 pp | Directly initialized |
| Mean PAQ-A score | 2.30 ± 0.70 | 2.3 | 0 | Matched baseline PA level |
| Low PA proportion | 37.40% | 37.40% | 0.00 pp | Directly initialized |
| Moderate PA proportion | 50.10% | 50.10% | 0.00 pp | Directly initialized |
| High PA proportion | 12.50% | 12.50% | 0.00 pp | Directly initialized |
| Average number of friends | 5.73 ± 4.57 | 6 | 0.27 | Rounded to nearest integer for network generation |
| Overall co-activity probability | 0.549 ± 0.200 | Used as calibration reference | NA | Used to derive calibrated per-step peer-influence process |
| Male co-activity probability | 0.585 ± 0.243 | Used as calibration reference | NA | Used to preserve sex-specific co-activity tendency |
| Female co-activity probability | 0.510 ± 0.231 | Used as calibration reference | NA | Used to preserve sex-specific co-activity tendency |
| PA target threshold | 2.75 | 2.75 | 0 | Literature-based stopping criterion |
| Note. PA = physical activity; PAQ-A = Physical Activity Questionnaire for Adolescents; pp = percentage points. The model initialization value refers to the baseline value used to initialize the ABM before intervention simulations. Survey-derived co-activity probabilities were used as empirical references for deriving the calibrated per-step peer-influence process rather than directly as per-step transition probabilities. | | | | |

**Supplementary Table 4.** PA level: distribution and group differences by gender in adolescents.

| PA-Level | Total(n=1692) | Male(n=877) | Female(n=815) | χ² | p |
| --- | --- | --- | --- | --- | --- |
| Low | 37.40% | 30.80% | 44.40% | 47.831 | 0.000** |
| Moderate | 50.10% | 52.60% | 47.50% |  |  |
| High | 12.50% | 16.60% | 8.10% |  |  |
| *indicates p<0.05, **indicates p<0.01. | | | | | |

**Supplementary Table 5.** PA level: distribution and group differences by grade in adolescents.

| PA-Level | Total (n=1692) | Grade 7 (n=561) | Grade 8 (n=874) | Grade9 (n=257) | *χ²* | *p* |
| --- | --- | --- | --- | --- | --- | --- |
| Low | 37.40% | 30.3% _a_ | 41.0% _b_ | 57.9% _c_ | 31.482 | **0.000^**^** |
| Moderate | 50.10% | 56.9% _a_ | 46.4% _b_ | 35.1% _b_ |  |  |
| High | 12.50% | 12.9% _a_ | 12.6% _a_ | 7.0% _a_ |  |  |
| Each subscript letter denotes a subset of grade categories. At the 0.05 level, the proportions in these categories do not differ significantly. (*indicates *p*<0.05, **indicates *p*<0.01). | | | | | | |

**Supplementary Table 6.** Social network nominations: differences by gender and grade in adolescents.

|  |  | Mean ± SD | *t* | *F* | *p* |
| --- | --- | --- | --- | --- | --- |
| Number of friends（n=1692） | Male (n=877) | 6.37 ± 5.167 | 6.087 | —— | **0.000^**^** |
|  | Female (n=815) | 5.05 ± 3.706 |  |  |  |
|  | Grade 7 (n=561) | 5.882 ± 4.88 | —— | 1.493 | 0.225 |
|  | Grade 8 (n=874) | 5.592 ± 4.56 |  |  |  |
|  | Grade 9 (n=257) | 6.438 ± 4.61 |  |  |  |
| Probability of exercising with friends (n=1692) | Male (n=877) | 0.585 ± 0.243 | 6.468 | —— | **0.000^**^** |
|  | Female (n=815) | 0.510 ± 0.231 |  |  |  |
|  | Grade 7 (n=561) | 0.553 ± 0.227 | —— | 0.378 | 0.685 |
|  | Grade 8 (n=874) | 0.547 ± 0.262 |  |  |  |
|  | Grade 9 (n=257) | 0.527 ± 0.23 |  |  |  |
| *indicates *p*<0.05, **indicates *p*<0.01. | | | | | |

**Supplementary Table 7.** Differences in steps between random intervention strategy and centrality-based strategies.

| Comparison | Random strategy, mean ± SD | Centrality-based strategy, mean ± SD | Mean difference | t value | p value | Interpretation |
| --- | --- | --- | --- | --- | --- | --- |
| Random vs Degree-centrality | 164.43 ± 24.18 | 155.13 ± 20.22 | 9.3 | 2.951 | 0.004 | Degree-centrality required fewer steps than random |
| Random vs Closeness-centrality | 164.43 ± 24.18 | 157.32 ± 17.13 | 7.11 | 2.4 | 0.017 | Closeness-centrality required fewer steps than random |
| Random vs Betweenness-centrality | 164.43 ± 24.18 | 153.45 ± 19.73 | 10.98 | 3.518 | 0.001 | Betweenness-centrality required fewer steps than random |
| Note. Values are mean ± SD simulation steps. Lower values indicate faster diffusion. Pairwise comparisons are intended as descriptive robustness checks rather than formal evidence that one centrality rule is universally superior. | | | | | | |

**Supplementary Table 8.** Sensitivity analysis of key model parameters

| Scenario | Parameter varied | Baseline value | Sensitivity value | Random | Degree-centrality | Closeness-centrality | Betweenness-centrality | Main interpretation |
| --- | --- | --- | --- | --- | --- | --- | --- | --- |
| Baseline | Original setting | — | — | 164.43 ± 24.18 | 155.13 ± 20.22 | 157.32 ± 17.13 | 153.45 ± 19.73 | Centrality-based strategies generally faster than random |
| S1 | PA target threshold | 2.75 | 2.65 | 158.26 ± 22.91 | 149.48 ± 18.86 | 151.37 ± 16.41 | 148.12 ± 18.94 | Centrality advantage generally retained |
| S2 | PA target threshold | 2.75 | 2.85 | 171.86 ± 25.36 | 162.94 ± 21.18 | 164.72 ± 18.35 | 160.67 ± 20.86 | Centrality advantage generally retained |
| S3 | Initial seed proportion | 0.15 | 0.12 | 173.62 ± 26.04 | 164.28 ± 21.76 | 166.11 ± 18.92 | 162.45 ± 21.54 | Centrality advantage generally retained |
| S4 | Initial seed proportion | 0.15 | 0.18 | 156.41 ± 22.35 | 147.36 ± 18.21 | 149.02 ± 15.96 | 146.25 ± 18.17 | Centrality advantage generally retained |
| S5 | Peer-influence process | 1.00 multiplier | 0.90 multiplier | 170.73 ± 25.11 | 161.45 ± 20.84 | 163.04 ± 18.02 | 159.86 ± 20.63 | Centrality advantage generally retained |
| S6 | Peer-influence process | 1.00 multiplier | 1.10 multiplier | 157.82 ± 22.73 | 148.96 ± 18.67 | 150.58 ± 16.24 | 147.73 ± 18.58 | Centrality advantage generally retained |
| Note. Values are mean ± SD simulation steps required to reach the population-level PA target. Lower values indicate faster diffusion. The peer-influence process was varied by multiplying the baseline calibrated setting by 0.90 and 1.10. | | | | | | | | |
